# Inclusion, Diversity, Equity, and Accessibility (IDEA) in multi-regional data research: An approach to facilitating change in a distributed research environment .

**DOI:** 10.23889/ijpds.v7i3.1934

**Published:** 2022-08-25

**Authors:** Amy Freier, Alan Katz

**Affiliations:** 1 Manitoba Centre for Health Policy

## Objectives

A recently established data research network identified the need to address Inclusion, Diversity, Equity, and Accessibility and established a team committed to IDEA informed change. The Team identifies, develops, and implements IDEA strategies in distributed research network. We will share the process of establishing this Team and outline identified coordinated opportunities moving forward.

## Approach

Administrative data and analyses are not neutral. Colonialism, racism, gender discrimination, ableism, and other forms of oppression have shaped the data that are available and the research processes that are used to analyze and evaluate data. It is imperative that health data research adopts and develops methods that embed IDEA. An informal survey of a health data research network identified four areas for action including capacity building, culture shifts, information sharing, and coordinated development of policies, practices, and tools. A multi-regional team was established to move forward with this work with an emphasis on operations and research practices.

## Results

In the fall of 2021 IDEA Team members were recruited from data centres across the network, including IDEA professionals, researchers, data collection and curation specialists, human resource professionals, and public/patient engagement specialists. The first IDEA Team meetings focused on team buildings and building shared purpose by shaping the Terms of Reference and creating principles for working together. Next steps will include an environmental audit to assess network capacity and a consensus oriented decision-making process to identify priorities. The Team includes over 20 members with a broad range of knowledge and expertise, making facilitation a key operational component. Establishing a baseline of knowledge and defining priorities will encompass the first year of the Teams work, navigating the distinct needs of a distributed network, the diverse needs of data centres, and the breadth of data that flows through the network.

## Conclusion

This multi-regional inter-disciplinary team is crucial to adopting, developing and embedding IDEA in a distributed data network and could serve as a model for other data research organizations. The Team will work towards capacity building, creating an internal culture shift and coordinating the development of new tools and resources.

